# Disagreement between Cardiac Troponin Tests Yielding a Higher Incidence of Myocardial Injury in the Emergency Setting

**DOI:** 10.3390/jcdd8030031

**Published:** 2021-03-23

**Authors:** Peter A. Kavsak, Shawn E. Mondoux, Janet Martin, Mark K. Hewitt, Lorna Clark, Nadia Caruso, Ching-Tong Mark, V. Tony Chetty, Craig Ainsworth, Andrew Worster

**Affiliations:** 1 Department of Pathology and Molecular Medicine, McMaster University, Hamilton, ON L8S 4L8, Canada; janet.martin@medportal.ca (J.M.); chetty@hhsc.ca (V.T.C.); 2 Hamilton Regional Laboratory Medicine Program, Juravinski Hospital, Hamilton, ON L8V 1C3, Canada; clarkl@hhsc.ca (L.C.); caruson@hhsc.ca (N.C.); markc@hhsc.ca (C.-T.M.); 3 Division of Emergency Medicine, Department of Medicine, McMaster University, Hamilton, ON L8S 4L8, Canada; shawn.e.mondoux@gmail.com (S.E.M.); mark.hewitt@medportal.ca (M.K.H.); worstea@mcmaster.ca (A.W.); 4 Division of Cardiology, Department of Medicine, McMaster University, Hamilton, ON L8S 4L8, Canada; ainswoc@mcmaster.ca

**Keywords:** high-sensitivity cardiac troponin, false positive, emergency department, myocardial injury

## Abstract

Differences in patient classification of myocardial injury between high-sensitivity cardiac troponin (hs-cTn) assays have largely been attributed to assay design and analytical sensitivity aspects. Our objective was to compare Ortho Clinical Diagnostics’ (OCD) hs-cTnI assay to OCD’s contemporary/conventional assay (cTnI ES) and another hs-cTnI assay (Abbott hs-cTnI) in samples obtained from different emergency departments (EDs). Two different sample types were evaluated (lithium heparin and ethylenediaminetetraacetic acid (EDTA) plasma) in a non-selected ED population (study 1, *n* = 469 samples) and in patients for which ED physicians ordered cardiac troponin testing (study 2, *n* = 1147 samples), from five different EDs. The incidence of injury in study 1 was higher with the OCD hs-cTnI assay (30.9%; 95% CI: 26.9 to 35.2) compared to that of the Abbott hs-cTnI (17.3%; 95% CI: 14.1 to 21.0) and the OCD cTnI ES (15.4%; 95% CI: 12.4 to 18.9) assays, with repeat testing identifying 4.8% (95% CI: 3.0 to 7.5) of the OCD hs-cTnI results with poor reproducibility. In study 2, 4.6% (95% CI: 3.5 to 6.0) of the results were not reported for the OCD hs-cTnI assay (i.e., poor reproducibility) with 12.7% (95%CI: 8.7 to 17.8) of the OCD hs-cTnI results positive for injury being negative for injury with the Abbott hs-cTnI assay. In summary, the OCD hs-cTnI assay yields higher rates of biochemical injury with a higher rate of poor reproducible results in different ED populations.

## 1. Introduction

In the emergency setting, physicians will order a cardiac troponin (cTn) test on patients presenting with symptoms suggestive of acute coronary syndrome (ACS) in order to rule-in or rule-out acute myocardial infarction. For newer versions of cTn tests, such as the high-sensitivity cardiac troponin (hs-cTn) assays, both analytical and clinical studies are required to verify their performance. Both diagnostic and prognostic studies in patients presenting with symptoms suggestive ACS in the emergency department (ED) are important for obtaining regulatory approval and supporting clinical adoption of newer tests such as the Ortho Clinical Diagnostics (OCD) hs-cTnI assay [1,2,3,4,5]. However, clinically, hs-cTnI is often measured in a broader group of patients in the ED to rule-out cardiac injury since elevated concentrations are associated with a multitude of cardiac conditions including ACS, tachyarrhythmias, and heart failure [6]. Within these elevated hs-cTnI samples, a small percentage of elevations are due to analytical issues/interferences, which are problematic for both the clinical and laboratory staff [7,8,9,10,11,12].

The Hamilton Regional Laboratory Medicine Program (HRLMP) provides laboratory services to five acute care hospitals: Hamilton General Hospital (HGH), Juravinski Hospital and Cancer Centre (JHCC), St. Joseph’s Healthcare Hamilton (SJHH), McMaster University Medical Centre and Children’s Hospital (MUMC), and West Lincoln Memorial Hospital (WLMH). Within a month of the HRLMP transitioning from the Abbott hs-cTnI assay to the OCD hs-cTnI assay, one of its hospitals identified quality control (QC) failures with the OCD hs-cTnI assay [13]. Subsequent work at the JHCC (hospital and cancer centre) and WLMH (small community hospital) indicated that the OCD hs-cTnI assay yielded a higher incidence of false positive results, in part due to an acute phase response [14,15,16]. To further understand the extent of patient misclassification with the OCD hs-cTnI assay in the emergency setting, two studies were undertaken to assess the sample type and the effect of patient selection on discordances for myocardial injury with different cTnI assays.

## 2. Materials and Methods

### 2.1. Study 1

ED patients from the five HRLMP hospital sites who had sufficient leftover de-identified lithium heparin plasma for testing (collected over 1 week from 6 October 2020 to 12 October 2020) were selected for additional testing. Briefly, aliquots (approximately 1 mL) of lithium heparin plasma (obtained within 8 h from collection) were frozen (−20 °C) and tested in batch mode at the JHCC site after appropriate pre-analytical handling (thawed at room temperature, mixed, centrifuged at 2300 *g* for 10 min). There were 469 samples (HGH = 137, JHCC = 150, SJH = 118, WLMH = 39, MUMC = 25) with sufficient volume for testing in the following order: Abbott ARCHITECT (Abbott Laboratories, Chicago, IL, USA) hs-cTnI, OCD (Ortho Clinical Diagnostics, Raritan, NJ, USA) VITROS XT 7600 hs-cTnI, OCD VITROS XT 7600 cTnI ES (run 1); then immediately retested in the following order except for cTnI ES (cTnI ES is OCD’s contemporary/conventional assay and in run 2 only hs-cTnI was measured to maximize the number of samples for duplicate measurements for the hs-cTnI assays *n* = 411). Acceptable repeats were identified if the difference between results was ≤3 ng/L for average concentrations <15 ng/L or ≤20% for concentrations ≥15 ng/L, consistent with previous analyses [12,16]. The mean, coefficient of variations (CVs), and number of measurements for the QC for the assays during the testing were as follows: OCD cTnI ES: level 1 mean = 0.076 µg/L/CV = 3.6%/*n* = 8; level 2 mean = 0.654 µg/L/CV = 2.5%/*n* = 8; level 3 mean = 7.168 µg/L/CV = 1.9%/*n* = 8 | OCD hs-cTnI: level 1 mean = 6.3 ng/L/CV = 13.7%/*n* = 25; level 2 mean = 36.5 ng/L/CV = 11.8%/*n* = 27; level 3 mean = 8120.9 ng/L/CV = 7.5%/*n* = 27 | Abbott hs-cTnI level 1 mean = 12.4 ng/L/CV = 4.1%/*n* = 9; level 2 mean = 49.2 ng/L/CV = 4.7%/*n* = 9; level 3 mean = 14,094.4 ng/L/CV = 12.9%/*n* = 12. The QC material for the OCD cTnI ES assay was from the assay manufacturer, whereas Thermo Fisher (Thermo Fischer Scientific, Waltham, MA, USA) Omni QC material was used for both the Abbott hs-cTnI and the OCD hs-cTnI assays at the JHCC [17].

### 2.2. Study 2

Performed in real-time and after study 1, it was decided for patient safety that all OCD hs-cTnI requests from the EDs from the five sites would be performed in duplicate. Only duplicate results with acceptable repeats (≤3 ng/L or ≤20%) had the average result reported, with poor repeat measurements not reported for the OCD hs-cTnI assay with the sample sent immediately for testing with the Abbott hs-cTnI assay [12,16]. Samples yielding a concentration greater than the upper reference limit (URL; i.e., >99th percentile cutoff from a healthy population, which indicates myocardial injury) for the OCD hs-cTnI assay (female 99th percentile cutoff ≤9 ng/L and male 99th percentile cutoff ≤13 ng/L) were also sent/reflexed for Abbott hs-cTnI testing at the JHCC (inpatient hs-cTnI testing for all hospitals was also performed with the Abbott hs-cTnI assay at the JHCC with female 99th percentile ≤16 ng/L and male 99th percentile ≤34 ng/L) [2,10]. The sample type was ethylenediaminetetraacetic acid (EDTA) plasma, which has been validated for both the OCD and Abbott hs-cTnI assays [2,3,4,18]. As part of the quality assurance process, we assessed the difference in myocardial injury between the OCD hs-cTnI and the Abbott hs-cTnI assays from the first week of this testing in the EDs (15 October 2020 to 21 October 2020). For all sites, the imprecision targets using Thermo Omni controls for the OCD hs-cTnI assay (level 1 ~5 ng/L, level 2 ~34 ng/L, level 3 ~7800 ng/L) was a SD ≤ 0.8 ng/L for level 1 and a CV ≤ 10% for levels 2 and 3, whereas for the Abbott hs-cTnI assay with the same controls (level 1 ~13 ng/L, level 2 ~51 ng/L, level 3 ~15,000 ng/L), the imprecision target was ≤10% for levels 1 through 3 in agreement with laboratory recommendations [19]. Chart reviews were performed for patients where OCD hs-cTnI concentrations were higher than Abbott hs-cTnI concentrations, which were all within the normal range (i.e., concentrations <99th percentiles).

### 2.3. Statistical Analyses

Passing-Bablok, Spearman correlation, and Kappa analyses were performed with the overall population 99th percentile cutoffs employed (Ortho hs-cTnI ≤ 11 ng/L, Abbott hs-cTnI ≤ 26 ng/L, and Ortho cTnI ES ≤ 0.034 µg/L) from the non-select ED population as no patient identifiers or demographics were obtained for study 1. The overall 99th URLs are recommended for the contemporary/conventional cTnI assays [6,19], with the overall cutoff still being recommended by some cardiology societies for the hs-cTn assay [20,21]. For study 2, sex-specific 99th percentiles were used to identify injury (as recommended by the Fourth Universal Definition of Myocardial Infarction) [6], with non-parametric analyses (i.e., Passing-Bablok, Spearman) performed with the 95% confidence intervals (CIs) derived for all estimates. Previous studies have documented that the OCD hs-cTnI assay yields lower concentrations (approximately 30% to 50% lower) as compared to the Abbott hs-cTnI assay [5,10], therefore, specific analyses were performed in the group where OCD hs-cTnI was higher than Abbott hs-cTnI; as this may indicate the presence of an interference [16]. Here, chart reviews were performed for those patients with normal Abbott hs-cTnI concentrations whose corresponding OCD hs-cTnI concentrations were higher than Abbott’s values. For these analyses (performed with GraphPad prism, Analyse-it, and MedCalc software), any cTnI concentration <1 ng/L was converted to 0.9 ng/L, with the Ortho cTnI ES concentrations converted to ng/L to enable comparison with the hs-cTnI assays (which report in ng/L concentrations).

## 3. Results

**Figure 1:**
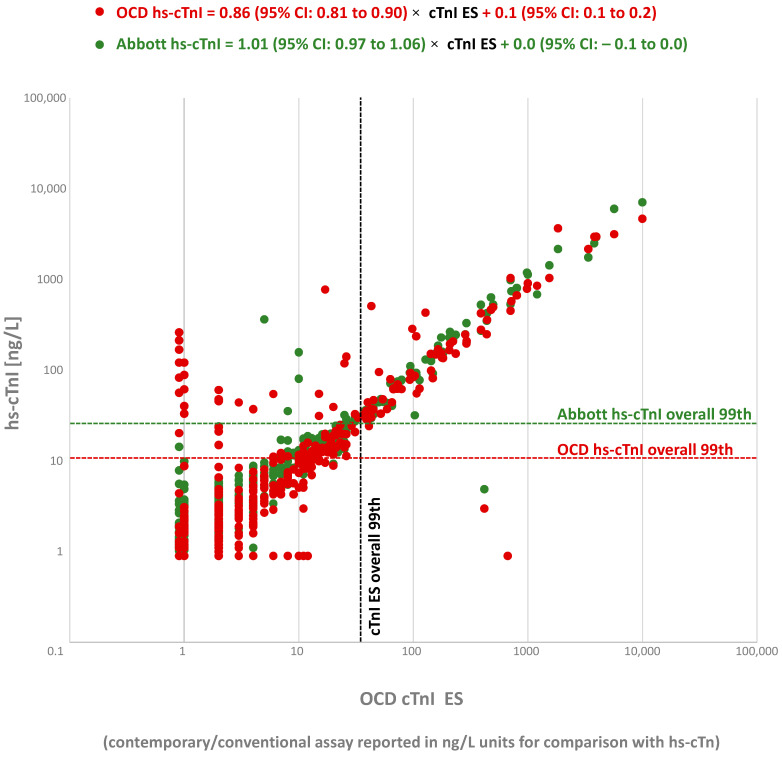
Comparison between the Ortho Clinical Diagnostics’ (OCD) hs-cTnI assay (red circles) and the Abbott hs-cTnI assay (green circles) versus the OCD cTnI ES assay in lithium heparin plasma. The regions where there is disagreement between assays regarding biochemical injury can be identified by the 99th percentile upper reference limit (ULR) cutoffs/dashed lines (black dashed line OCD cTnI ES 99th, red dashed line OCD hs-cTnI 99th, green dashed line Abbott hs-cTnI 99th).

In study 1, assessing the non-select ED population with lithium heparin plasma, the overall agreement between the Abbott hs-cTnI and the OCD cTnI ES concentrations (*n* = 469; slope = 1.01; 95% CI: 0.97 to 1.06/rho = 0.89; 95% CI: 0.87 to 0.91/Kappa = 0.90; 95% CI: 0.84 to 0.95; 97.3% agreement) was higher as compared to that of the OCD hs-cTnI and the OCD cTnI ES assays (*n* = 469; slope = 0.86; 95% CI: 0.81 to 0.90/rho = 0.82; 95% CI:0. 79 to 0.85/Kappa = 0.55; 95% CI: 0.47 to 0.64; 83.6% agreement) (Figure 1). In this non-select ED population, biochemical injury was significantly higher for the OCD hs-cTnI assay (30.9%; 95% CI: 26.9 to 35.2) as compared to that of the Abbott hs-cTnI assay (17.3%; 95% CI: 14.1 to 21.0) and the OCD cTnI ES assay (15.4%; 95% CI: 12.4 to 18.9). Further comparisons between the hs-cTnI assays below 100 ng/L for the OCD cTnI ES assay (*n* = 426) yielded similar estimates (i.e., Passing-Bablok regression and Spearman correlation for Abbott hs-cTnI = 1.05 (95% CI: 1.00 to 1.13) × OCD cTnI ES—0.04 (95% CI: −0.11 to 0.00) and rho = 0.87; 95% CI: 0.84 to 0.89 | for OCD hs-cTnI = 0.90 (95% CI: 0.85 to 0.95) × OCD cTnI ES + 0.09 (95% CI: 0.05 to 0.14) and rho = 0.79; 95% CI: 0.75 to 0.82). In the 411 samples with sufficient volume for repeat testing, the OCD hs-cTnI assay yielded 4.8% (95% CI: 3.0 to 7.5), results with poor reproducibility as compared to 2.2% (95% CI: 1.0 to 4.2) with the Abbott hs-cTnI assay (*p* = 0.041; Table 1 lists the values yielding poor reproducibility).

**Figure 2:**
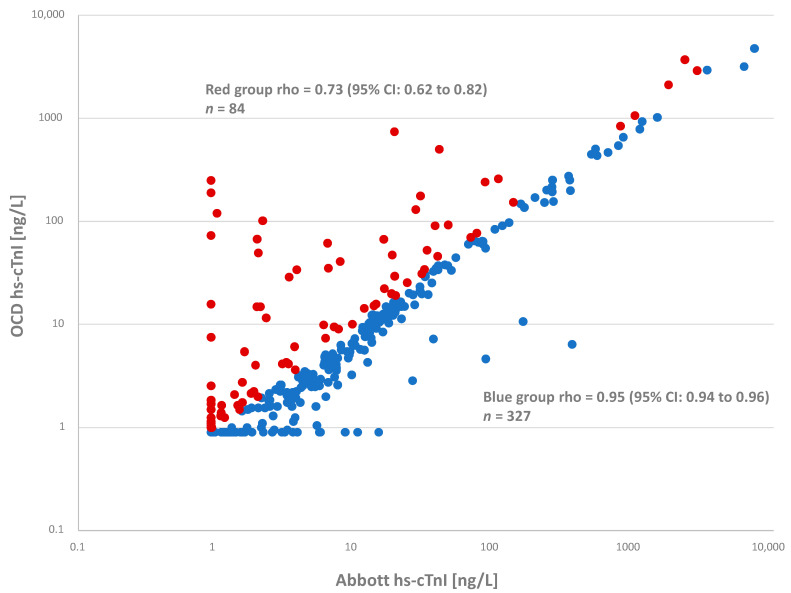
Correlation between the OCD hs-cTnI versus Abbott hs-cTnI assay using the average concentrations from the 411 lithium heparin plasma samples (Spearman rho = 0.87; 95% CI: 0.84 to 0.91). The red full circles (*n* = 84) are paired results where OCD hs-cTnI is higher than Abbott hs-cTnI (Spearman rho = 0.73; 95% CI: 0.62 to 0.82) with the blue full circles (*n* = 327) being paired results where OCD hs-cTnI is not higher than Abbott hs-cTnI (Spearman rho = 0.95; 95% CI: 0.94 to 0.96).

Plotting the average OCD hs-cTnI versus the average Abbott hs-cTnI (*n* = 411) results yielded the following equation: OCD = 0.81 (95% CI: 0.77 to 0.85) × Abbott hs-cTnI + 0.2 (95% CI: 0.1 to 0.2) with a correlation (rho) of 0.87 (95% CI: 0.84 to 0.91) with 20.4% (*n* = 84) paired results yielding higher concentrations for the OCD hs-cTnI assay. The percentage of injury in this group (*n* = 84) was 55.9% (95% CI: 41.1 to 74.4) for the OCD hs-cTnI assay as compared to only 22.6% (95% CI: 13.6 to 35.3) with the Abbott hs-cTnI assay (*p* < 0.01). Removal of these 84 samples increased the correlation between OCD hs-cTnI and Abbott hs-cTnI (rho = 0.95; 95% CI: 0.94 to 0.96) and yielded the following equation: OCD = 0.72 (95% CI: 0.69 to 0.75) × Abbott hs-cTnI + 0.3 (95% CI: 0.1 to 0.3) (*n* = 327) (Figure 2).

**Figure 3:**
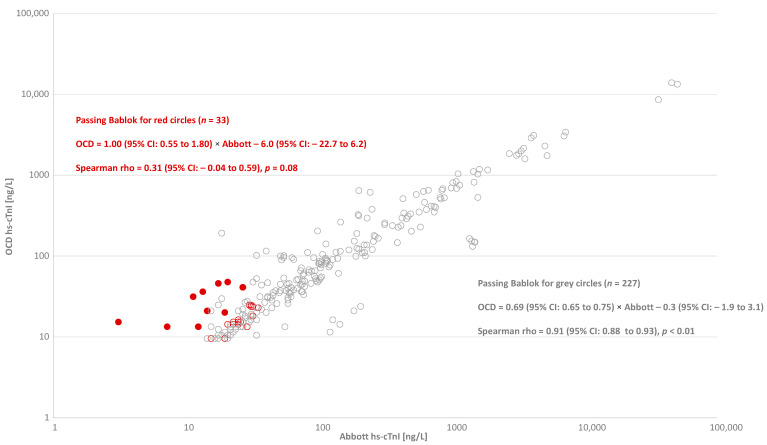
In 260 ethylenediaminetetraacetic acid (EDTA) plasma samples from the emergency department (ED) that yielded results for OCD hs-cTnI above the sex-specific 99th percentile URL cutoffs (i.e., positive results), the correlation between OCD hs-cTnI and Abbott hs-cTnI in samples where Abbott hs-cTnI values are above the URLs is indicated by the grey open circles (*n* = 227). The red circles represent paired results where Abbott hs-cTnI levels are normal (*n* = 33), with the red full circles representing paired results where OCD hs-cTnI is higher than Abbott hs-cTnI (*n* = 10).

In study 2, physicians ordered/requested measurements on 1147 samples (HGH = 491, JHCC = 303, SJH = 281, WLMH = 65, MUMC = 7 samples) from 721 patients in the EDs. There were 53 samples that were not reported for the OCD hs-cTnI assay (i.e., poor reproducibility) for an incidence error/non-reported rate of 4.6% (95% CI: 3.5 to 6.0). These samples were re-tested for the Abbott hs-cTnI assay, of which 51% yielded normal concentrations with the Abbott hs-cTnI assay. There were 260 samples yielding an average OCD hs-cTnI concentration >99th percentile URL cutoffs that reflexed and had Abbott hs-cTnI results. The 260 samples were further classified by those samples that yielded normal Abbott hs-cTnI results (*n* = 33 or 12.7%; 95%CI: 8.7 to 17.8) and those whose hs-cTnI concentrations were indicative of injury (*n* = 227 or 87.3%; 95%CI: 76.3 to 99.4). The correlation between OCD hs-cTnI and Abbott hs-cTnI was significantly higher (rho = 0.91; 95% CI: 0.88 to 0.93) in samples where Abbott hs-cTnI indicated injury (*n* = 227) as compared to those indicating no injury (*n* = 33 and rho = 0.31; 95% CI: −0.04 to 0.59) (Figure 3). Of the 33 samples with normal Abbott hs-cTnI concentrations, 10 samples had higher OCD hs-cTnI concentrations as compared to those from Abbott hs-cTnI. These 10 samples were from 8 patients without a diagnosis of ACS (the primary diagnoses were related to trauma, falls, pneumonia, pleural effusion, heart failure, and COVID-19).

## 4. Discussion

The findings from both studies reported here support recent clinical data indicating that the OCD hs-cTnI may misclassify biochemical injury in approximately 10% of the population being evaluated for myocardial injury [14,15,16]. Despite OCD using the same three antibodies in their cTnI ES (contemporary/conventional) assay and their hs-cTnI (high-sensitivity) assay [22], there is better agreement between the cTnI ES assay with the Abbott hs-cTnI assay as compared to the OCD hs-cTnI assay. Differences in assay design (i.e., capture versus detection antibody configuration or the antibody biotinylation process), incubation/time, washing, etc. might be possible variable(s) contributing to the higher and poorly reproducible results obtained with the OCD hs-cTnI assay in some patient samples. Specifically, patients with a primary diagnosis that can yield high acute phase reactants appear to be a group that is susceptible to the OCD hs-cTnI assay yielding false positive results, as evident from these data and other publications [14,15,16]. OCD has confirmed these findings of certain samples yielding high and non-reproducible results and is conducting studies to further understand this matter.

There is no standardization for hs-cTn assays; hence, different assays will yield different results and, importantly, different URLs (i.e., assay-specific) need to be utilized to identify myocardial injury [19,23]. Some have suggested that the differences observed between cTn assays may in part be due to macrocomplex detection [24,25]. Indeed, macrocomplexes often explain why Abbott hs-cTnI levels are several-fold higher than OCD hs-cTnI concentrations [26], but not necessarily why OCD hs-cTnI levels will be higher than Abbott hs-cTnI [10,14,15]. Here, we have recently reported that patients with an acute phase response may yield high and non-reproducible results with the OCD hs-cTnI assay [16]. Importantly, we have extended these findings by assessing the incidence of “false positive” results with the OCD hs-cTnI assay across different ED populations and with different plasma sample types that are used in the acute setting to provide fast turnaround times. Removal of the paired results where OCD hs-cTnI concentrations were higher than Abbott hs-cTnI (i.e., the potential “false positive” OCD values), resulted in higher correlations with Abbott hs-cTnI (rho > 0.90), with the overall relationship (i.e., slope by regression analyses) similar to what has been previously reported for these assays (i.e., with OCD hs-cTnI having ~30% lower values than Abbott hs-cTnI). Other contributing factors that may also explain differences in the incidence of myocardial injury are related to the derivation of the 99th percentile URLs, where the selection criteria of the healthy population and statistical techniques utilized may yield very different 99th percentile URLs within and between the hs-cTn assays [23,27]. However, until more information is available, caution is warranted for interpreting the OCD hs-cTnI assay in the emergency setting as the common pre-analytical variables contributing to discordant results [7,8,9,10,11,12] does not appear to be the main factor causing these discordant and non-reproducible results for ED patients.

## Figures and Tables

**Table 1:** Lithium heparin plasma samples that yielded poor reproducibility results (ng/L) for the OCD hs-cTnI assay and Abbott hs-cTnI assay. The bolded values indicate where both OCD hs-cTnI results are higher than the Abbott hs-cTnI results.

OCD Poor Reproducibility Results						
OCD hs-cTnI 1st	OCD hs-TnI 2nd	Average	Difference between OCD Results	OCD TnI-ES	Abbott hs-cTnI 1st	Abbott hs-cTnI 2nd
**8.1**	**11.7**	9.9	3.6	5	6.1	5.5
**8.6**	**14.6**	11.6	6	2	1.7	2.8
**4.4**	**10.7**	7.6	6.3	0.9	0.9	0.9
**8.8**	**2.1**	5.5	6.7	1	1.8	1.3
**47.0**	**58.3**	52.7	21%	45	32.6	31.6
**168.1**	**210.9**	189.5	23%	0.9	0.9	0.9
**83.3**	**62.6**	73.0	28%	0.9	0.9	0.9
**33.2**	**24.4**	28.8	31%	1	3.2	3.3
**39.4**	**55.2**	47.3	33%	20	18.3	17.8
**55.1**	**79.1**	67.1	36%	15	15.1	16.3
**286.9**	**195.8**	241.4	38%	98	83.6	84.4
**121.3**	**81.7**	101.5	39%	0.9	2.0	2.3
**141.8**	**213.2**	177.5	40%	26	29.2	28.6
**20.3**	**11.1**	15.7	59%	0.9	0.9	0.9
**44.2**	**24.0**	34.1	59%	3	3.5	3.9
**89.0**	**45.7**	67.4	64%	1	1.7	2.1
**54.9**	**26.7**	40.8	69%	6	7.0	8.2
**48.2**	**22.2**	35.2	74%	2	6.5	6.1
**37.3**	**85.7**	61.5	79%	4	6.0	6.4
**21.2**	**8.5**	14.9	86%	2	1.9	2.1
**Abbott Poor Reproducible Results**						
**Abbott hs-cTnI 1st**	**Abbott hs-cTnI 2nd**	**Average**	**Difference between Abbott Results**	**OCD TnI-ES**	**OCD hs-cTnI 1st**	**OCD hs-TnI 2nd**
2514.3	3115.7	2815.0	21%	3790	2958.0	2864.0
2938.5	3704.9	3321.7	23%	3950	2975.0	2924.0
31.9	41.5	36.7	26%	103	87.2	94.6
687.6	897.6	792.6	26%	1200	854.1	830.0
273.4	398.6	336.0	37%	390	279.5	271.8
206.3	302.6	254.4	38%	292	209.3	225.7
40.6	62.8	51.7	43%	65	44.5	44.5
12.1	18.8	15.5	43%	20	8.9	8.0
15.4	25.1	20.2	48%	19	16.5	16.8

## Data Availability

The data presented in this study are available on reasonable requests from the corresponding author. The data are not publicly available due to privacy.
